# Spatial multimodal analysis of transcriptomes and metabolomes in tissues

**DOI:** 10.1038/s41587-023-01937-y

**Published:** 2023-09-04

**Authors:** Marco Vicari, Reza Mirzazadeh, Anna Nilsson, Reza Shariatgorji, Patrik Bjärterot, Ludvig Larsson, Hower Lee, Mats Nilsson, Julia Foyer, Markus Ekvall, Paulo Czarnewski, Xiaoqun Zhang, Per Svenningsson, Lukas Käll, Per E. Andrén, Joakim Lundeberg

**Affiliations:** 1 Department of Gene Technology, KTH Royal Institute of Technology, Science for Life Laboratory, Stockholm, Sweden; 2 Department of Pharmaceutical Biosciences, Spatial Mass Spectrometry, Science for Life Laboratory, Uppsala University, Uppsala, Sweden; 3 Department of Biochemistry and Biophysics, Stockholm University, Science for Life Laboratory, Stockholm, Sweden; 4 Department of Clinical Neuroscience, Section of Neurology, Karolinska Institutet, Stockholm, Sweden; 5 Basic and Clinical Neuroscience, King’s College London, London, UK

**Keywords:** Genome-wide analysis of gene expression, Mass spectrometry, Parkinson's disease, Transcriptomics, Metabolomics

## Abstract

We present a spatial omics approach that combines histology, mass spectrometry imaging and spatial transcriptomics to facilitate precise measurements of mRNA transcripts and low-molecular-weight metabolites across tissue regions. The workflow is compatible with commercially available Visium glass slides. We demonstrate the potential of our method using mouse and human brain samples in the context of dopamine and Parkinson’s disease.

## Abstract (web summary)

Metabolites and RNA in a tissue section are profiled simultaneously.

## Main

Spatially resolved transcriptomics (SRT) allows the measurement of genome-wide mRNA expression and provides positional information about the mRNA in a tissue section. Although key aspects of SRT technologies can vary, each technique will ultimately yield a gene-expression count table with tissue coordinates^1–3^. Mass spectrometry imaging (MSI) enables label-free, spatially resolved measurement of the abundance of biomolecules directly from fresh frozen tissue sections^4,5^. In matrix-assisted laser desorption/ionization (MALDI)-MSI, a matrix is applied to the surface of tissue sections mounted onto a glass slide. Focusing a pulsed laser beam onto the tissue section then generates ionic species from the molecules present in the sample surface, enabling the collection of mass-to-charge (*m/z*) spectra at defined raster positions from across the tissue section. Although the SRT and MSI technologies are becoming more popular in spatial biology, they are currently applied as separate methodologies due to experimental constraints such as noncharged, barcoded (in SRT) versus conductive (in MALDI-MSI) microscopy slides, along with the risk of RNA degradation during the harsh MSI process^6,7^. In the present study, we demonstrate the possibility of combining SRT and MALDI-MSI in a single tissue section with retained specificity and sensitivity of both modalities by introducing a spatial multimodal analysis (SMA) protocol.

**Fig. 1:**
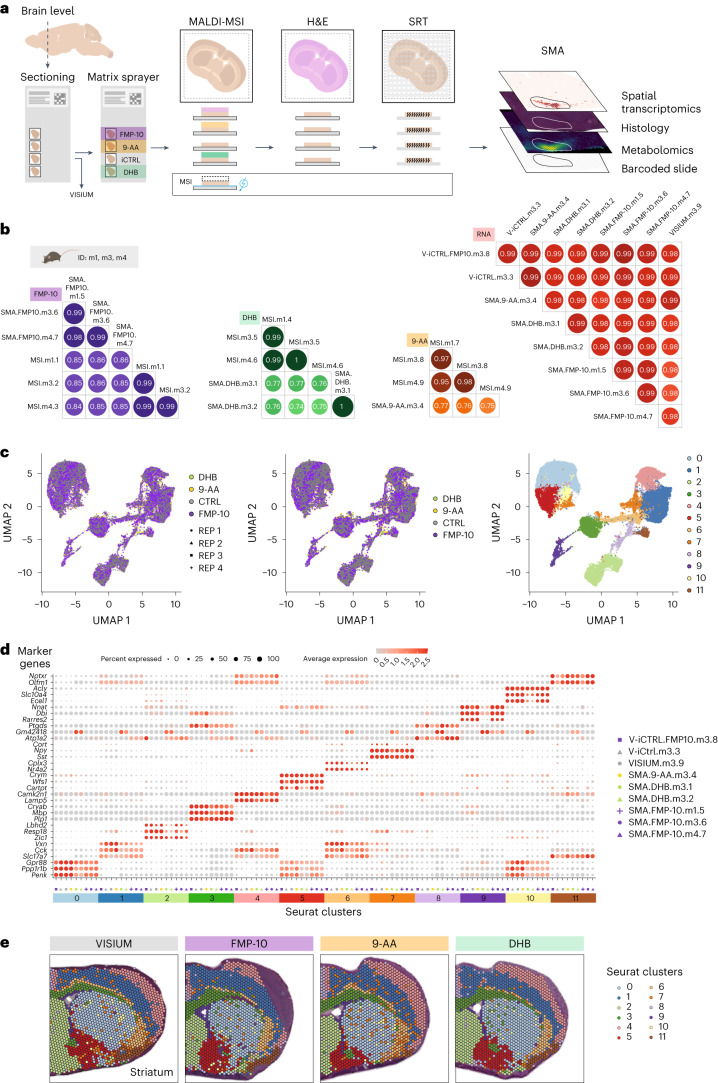
A multimodal spatial omics approach to investigate metabolites, morphology and gene expression analysis. **a**, The SMA workflow and quality control design—nonembedded, snap-frozen samples are sectioned and thaw-mounted onto noncharged, barcoded Visium Gene Expression arrays. Tissue sections are then sprayed with MALDI matrices and MSI is performed. This is followed by H&E staining and imaging with bright field microscopy. Finally, sections are processed for SRT. We also designed the following three types of control samples: (1) MSI—samples processed with standard MALDI-MSI protocol on ITO conductive slides; (2) VISIUM—samples processed with standard Visium protocol on all four capture areas of a Visium Gene Expression array and (3) V-iCTRL—samples processed with Visium protocol, but MALDI-MSI was performed on other capture areas of a Visium Gene Expression array. **b**, Pairwise gene-to-gene and molecule-to-molecule correlations across biological replicates. Samples are named with short identifiers that reflect the technical conditions under which the sample was analyzed: MSI, stand-alone MALDI-MSI; SMA, SMA protocol; VISIUM, stand-alone Visium. Additional acronyms indicate the matrix used in the SMA protocol (FMP-10, DHB and 9-AA), the sample (m1, m3 or m4) and the serial number of the tissue section (one to nine for each section placed on either ITO or Visium slides). **c**, UMAP of SMA ST spots colored by sections (left), MALDI matrices (middle) and clusters (right). **d**, Top three marker genes with highest average log_2_ fold change for each spatial cluster across biological replicates. **e**, Spatial plot of mouse brain tissue sections (striatal level, 0.49 mm from bregma) that illustrates clusters of transcripts for samples sprayed with three different MALDI matrices (FMP-10, 9-AA and DHB) and one sample processed with the stand-alone Visium protocol.

**Extended Data Fig. 1:**
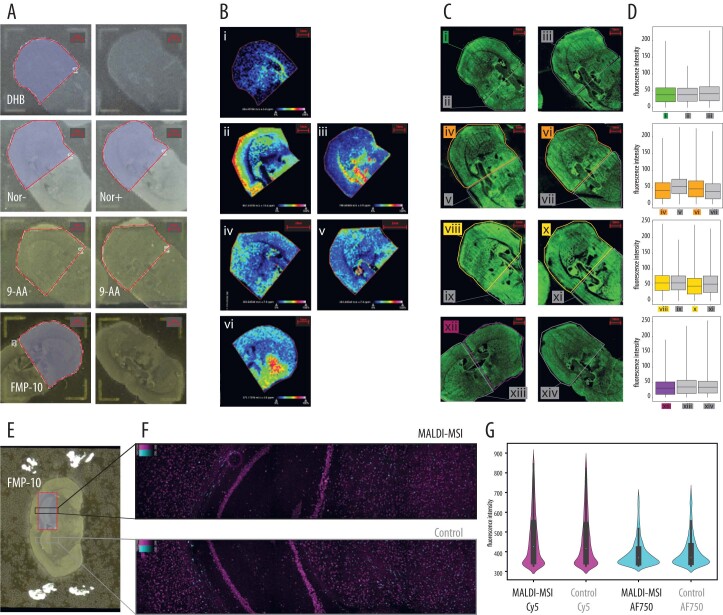
SMA using four different MALDI matrices. **(a)** Eight mouse brain tissue sections from the striatal level of the same animal (n = 8) were mounted onto a Visium Tissue Optimization slide and sprayed with four different MALDI matrices (DHB, norharmane (analyzed in both positive and negative mode, shown as Nor+ and Nor-), 9-AA and FMP-10). Areas delimited by red lines: regions of interest imaged with MALDI-MSI. Scalebars: 1 mm. **(b)** Representative MSI results from: i) *m/z* 426.36, C18:1 L-Carnitine (DHB); ii) *m/z* 857.52, PI(36:4) (Nor-); iii) *m/z* 788.62 PC(36:1) (Nor+); iv,v) *m/z* 303.24, arachidonic acid (9-AA); vi) *m/z* 371.17, GABA (FMP-10). Nor+ and Nor-: Norharmane analyzed in positive and negative mode, respectively. Scalebars: 1 mm, except iv and v where it is 2 mm. **(c)** Fluorescence microscopy images of mRNA footprint captured with polydT probes after MALDI-MSI. Colored lines (i, iv, vi, viii, x, xii) demarcate areas imaged with MALDI-MSI, while gray lines (ii, iii, v, vii, ix, xi, xiii, xiv) demarcate areas not imaged with MALDI-MSI and used as controls. Scalebars: 1 mm. **(d)** Fluorescence intensity of tissue areas imaged or not with MALDI-MSI. The upper and lower limit of the box represent the +1 and −1 standard deviation from the mean, the horizontal line inside the box represents the mean fluorescence intensity, and the upper and lower limits of the whiskers represent the maximum and minimum fluorescence intensity values. The results shown in panels (A-C) belong to eight consecutive tissue sections from n = 1 biologically independent sample examined over one independent experiment (all the sections were placed on one Visium Tissue Optimization array). The areas in square pixels over which the statistics is derived are the following: i = 768047, ii=355349, iii=843707, iv=866085, v = 578711, vi=805789, vii=562179, viii=846042, ix=317398, x = 843416, xi=611982, xii=779667, xiii=727089, xiv=751797. **(e)** A mouse brain tissue sections (n = 1) from the hippocampus level was mounted onto an ITO slide and sprayed FMP-10. The area delimited by a red line demarcates the region of interest imaged with MALDI-MSI. **(f)** Targeted In Situ Sequencing data demonstrate similar rolling circle product (RCP) density generated from MALDI-MSI processed region (upper right panel) and non-processed region (lower right panel) for demarcated regions of interest in the mouse coronal section (n = 1). Targeted ISS simultaneously probed for housekeeping gene, *Gapdh* labeled in Magenta (Cy5), and a panel of five control genes - *Foxj1, Plp1, Lamp5, Rorb* and *Kcnip2* that are labeled in Cyan (AF750). **(g)** Mean Cy5 and AF750 fluorescence intensity of rolling circle products in tissue areas imaged or not with MALDI-MSI.The results shown in panels (E-G) belong to one tissue section from n = 1 biologically independent sample examined over one independent experiment. The number of RCPs detected in the MALDI-MSI processed region in AF750 and Cy5 and the number of RCPs detected in the non-processed region in AF750 and Cy5 respectively, which the statistics is derived from, are the following: n = 3830,n = 18231, n = 3051,n = 18193. The lower and upper hinges of the boxplot correspond to the first and third quartiles (the 25th and 75th percentiles), the central white dot corresponds to the median, the upper and lower whiskers extend from the hinge to the maximum or minimum respectively.

**Extended Data Fig. 2:**
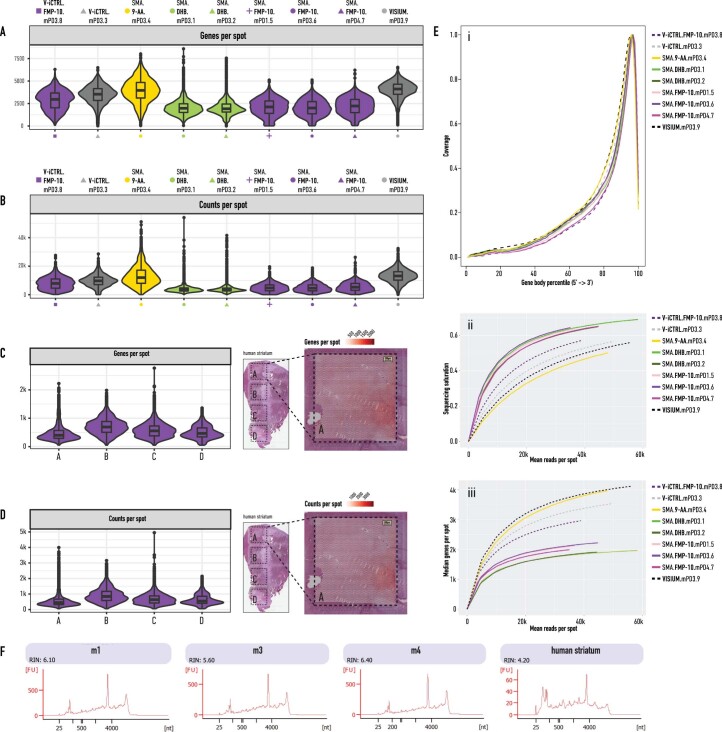
Spatial transcriptomics data quality control. Violin plots and box plots illustrating the number of unique genes per spot **(a)** and the number of unique molecular identifiers (UMIs) per spot **(b)** across biological conditions of the mouse striatum data (n = 9). The numbers of spots per section from which the statistics is derived are the same for the corresponding sections in panels A and B, and are the following: V-iCTRL.FMP10.mPD3.8 = 3017, V-iCTRL.nM.mPD3.3 = 3163, SMA.9AA.mPD3.4 = 2913, SMA.DHB.mPD3.1 = 2856, SMA.DHB.mPD3.2 = 3002, SMA.FMP10.mPD1.5 = 2675, SMA.FMP10.mPD3.6 = 3120, SMA.FMP10.mPD4.7 = 2918, VISIUM.mPD3.9 = 3116. n = 9 sections examined over 3 biologically independent samples. Violin plots and box plots illustrating the number of unique genes per spot **(c)** and the number of unique molecular identifiers (UMIs) per spot **(d)** of the human striatum data (n = 1). The human sample H&E was used as a legend to indicate the four capture areas A-D. The numbers of spots per capture area from which the statistics is derived are the same for corresponding sections in panels C and D and are the following: A = 4770, B = 4875, C = 4740, D = 4387. n = 4 capture areas examined over 1 biologically independent sample. For all boxplots presented in (A-D) the lower and upper hinges of the boxplot correspond to the first and third quartiles (the 25th and 75th percentiles), the central line corresponds to the median, the upper and lower whiskers extend from the hinge to the largest or smallest value respectively no further than 1.5 times the inter-quartile range, data beyond the end of the whiskers are plotted individually as black dots. On the right, spatial featureplot representing the number of genes per spot and the number of UMIs per spot of a representative capture area (that is, capture area A). **(e)** Sequencing metrics: i) Gene body coverage plot illustrating the sequencing coverage at different percentiles of gene body for all the genes in the quality control dataset; ii) sequencing saturation as a function of mean reads per spot; iii) median genes per spot as a function of mean reads per spot. **(f)** RNA integrity plots of mouse and human post-mortem samples.

The SMA workflow comprises the following four steps: (1) sectioning nonembedded snap-frozen samples onto noncharged, barcoded gene expression arrays, (2) MSI by MALDI, (3) hematoxylin and eosin (H&E) staining and bright field microscopy and (4) SRT (Fig. 1a). The SMA workflow does not require any modifications to the commercially available Visium glass slides, nor modifications to the MALDI-MSI or SRT protocols, except for three washes in cold methanol at the end of step 2 to wash away the matrix. To test the feasibility of our method, we assessed whether RNA was still present after tissue exposure to MALDI-MSI by using a slide coated with polydT probes. We mounted coronal sections of a mouse brain and sprayed it with the following four different MALDI matrices: (1) 9-aminoacridine (9-AA) for detection of metabolites in negative ionization mode, (2) 2,5-dihydroxybenzoic acid (DHB) for detection of metabolites in positive ionization mode, (3) norharmane for detection of various lipids and (4) 4-(anthracen-9-yl)-2-fluoro-1-methylpyridin-1-ium iodide (FMP-10), which charge-tags molecules with phenolic hydroxyls and/or primary amines, including neurotransmitters^8^. We imaged the sections using Fourier-transform ion cyclotron resonance–MALDI MSI and collected spectra for approximately 3 h at room temperature. Fluorescence microscopy imaging of the cDNA footprint generated during the Tissue Optimization assay (Methods) showed that the captured transcripts correlated well with tissue morphology, indicating that mRNA, surprisingly, is still present after MALDI-MSI in all of the investigated matrices (Extended Data Figs. 1a–c and 2). The presence of mRNA post-MALDI was confirmed using targeted in situ sequencing^9^ on coronal mouse sections with FMP-10 on conductive MALDI slides (Extended Data Fig. 1d).

**Extended Data Fig. 3:**
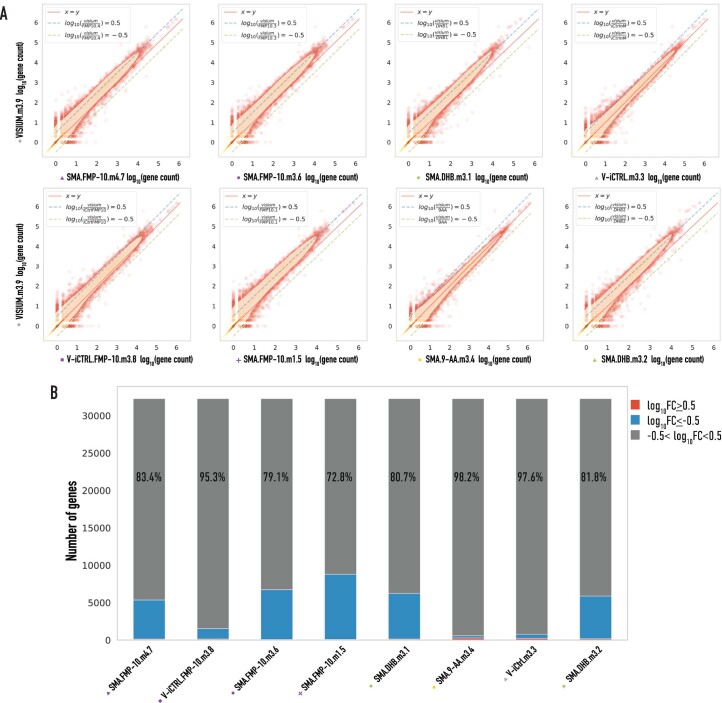
Performance analysis of SMA-SRT data. **(a)** Scatterplots of log_10_ gene counts of SMA-SRT data vs. stand-alone Visium data. The red line highlights a 1-to-1 relationship, whereas the dashed green and blue lines highlight a log_10_ 0.5 or −0.5 relationship. **(b)** Stacked barplot illustrating the percentage of genes with log_10_ higher, lower or within the log_10_ fold change range −0.5-0.5. The percentages inside the gray bars illustrate the percentages of peaks with absolute log_10_ below 0.5.

**Extended Data Fig. 4:**
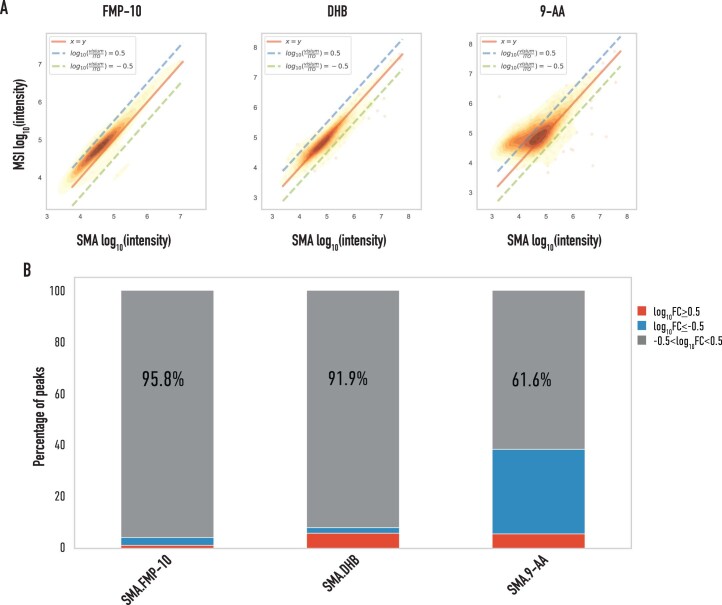
Performance analysis of SMA-MSI data. **(a)** Scatterplots of log_10_ peak intensities of SMA-MSI data vs stand-alone MALDI-MSI data. The red line highlights a 1-to-1 relationship, whereas the dashed green and blue lines highlight a log_10_ 0.5 or −0.5 relationship. **(b)** Stacked barplot illustrating the percentage of peaks with log_10_ higher, lower or within the log_10_ fold change range −0.5-0.5. The percentages inside the gray bars illustrate the percentages of peaks with absolute log_10_ below 0.5.

Next, we investigated the reproducibility of SMA. For this purpose, we repeated the experiments using barcoded Visium oligonucleotide slides, which enabled the quantification of individual captured transcripts by sequencing. We used seven consecutive coronal mouse brain sections from three different mice, imaged with three different MALDI matrices (9-AA, DHB and FMP-10). We compared MALDI-MSI and gene expression data from matching tissue sections analyzed with MALDI-MSI or Visium, respectively. SMA data analysis demonstrated that the small molecule and gene expression profiles correlated well (*r* > 0.74) with the reference data (Fig. 1b). Furthermore, 61.6–98.2% of the molecules were expressed within an absolute log_10_ fold change below 0.5 compared to stand-alone MALDI-MSI or Visium (Extended Data Figs. 3 and 4), without appreciable effect on molecule identification and downstream biological analysis. Joint analysis and visualization of transcriptomics data showed that the gene expression integrates well across experimental conditions, with a similar expression of marker genes and high conservation of the spatial clusters (Fig. 1c–e). These results provide strong evidence that SMA can be used with several matrices for simultaneous gene expression and biomolecule profiling.

**Fig. 2:**
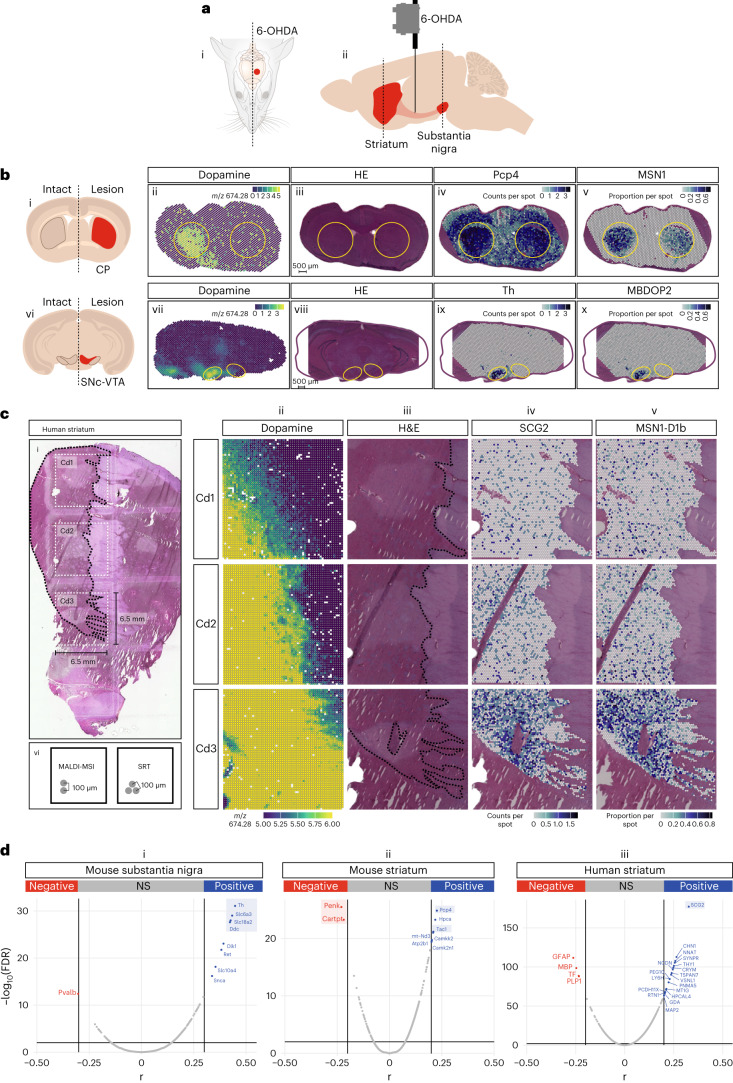
Spatial multimodal analysis of a Parkinson’s disease mouse model and a human postmortem brain affected by Parkinson’s disease. **a**, Cartoon showing the injection of 6-OHDA only in one hemisphere in the MFB. Dashed lines indicate the depth (0.49 and −3.39 mm, distance from bregma) for the substantia nigra and striatum, respectively. **b**, Representative sections from the substantia nigra and striatum of the mouse PD model. From left to right: cartoon showing the dopamine-depleted regions (i and vi), dopamine expression (ii and vii), H&E staining (iii and viii), spatial gene expression of the gene with the highest correlation to dopamine (iv and ix), proportions of MSN1 (v) and MBDOP2 (x). The remaining two striatal and two nigral sections processed with SMA and FMP-10 present in our dataset show similar results. **c**, Human postmortem striatum sample. From left to right: images are presented in the same order as in **b**. The demarcated area indicates the caudate nucleus of the striatum. See Fig. 2 for gene counts statistics. **d**, From left to right (i, ii and iii): dopamine-to-gene correlations in the mouse substantia nigra, mouse striatum and human striatum. Cd, caudate nucleus; CP, caudoputamen; NS, not significant.

**Extended Data Fig. 5:**
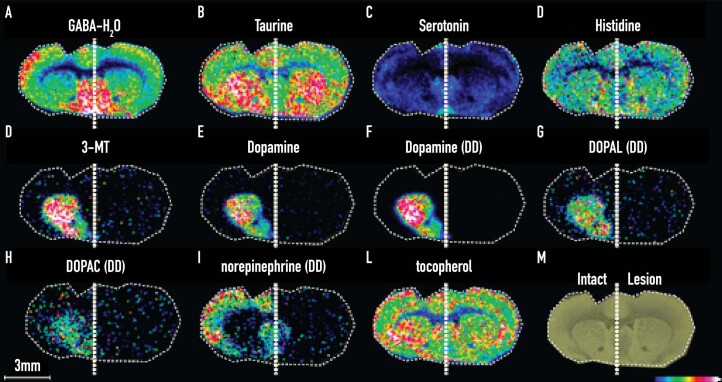
Selection of ion images from key neurotransmitters and metabolites in coronal tissue sections from a mouse PD model. Ion distribution images of **(a)** GABA-H_2_O, **(b)** taurine, **(c)** serotonin, **(d)** histidine, **(e)** 3-MT, **(f)** dopamine, **(g)** dopamine, double derivatized (DD), **(h)** DOPAL (DD), **(i)** DOPAC (DD), **(j)** norepinephrine (DD), **(k)** tocopherol, and **(l)** scanned image of the coronal mouse tissue section that was analyzed. All ion distribution images are scaled to 50% of max intensity and presented as single derivatized species, unless otherwise stated.

**Extended Data Fig. 6:**
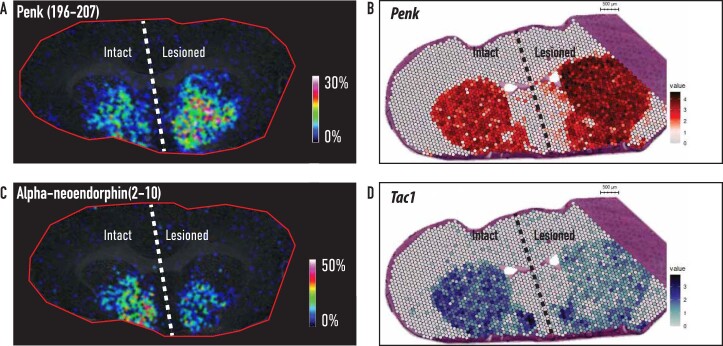
Expression of neuropeptides and corresponding genes in mouse striatum. Ion distribution images of **(a)** Penk(197-208) (SPQLEDEAKELQ, m/z 1386.67) and **(c)** Alpha-neoendorphin(2-10) (GGFLRKYPK, m/z 1065.62). Spatial gene expression of **(b)** *Penk* and **(d)** *Tac1*.

**Extended Data Fig. 7:**
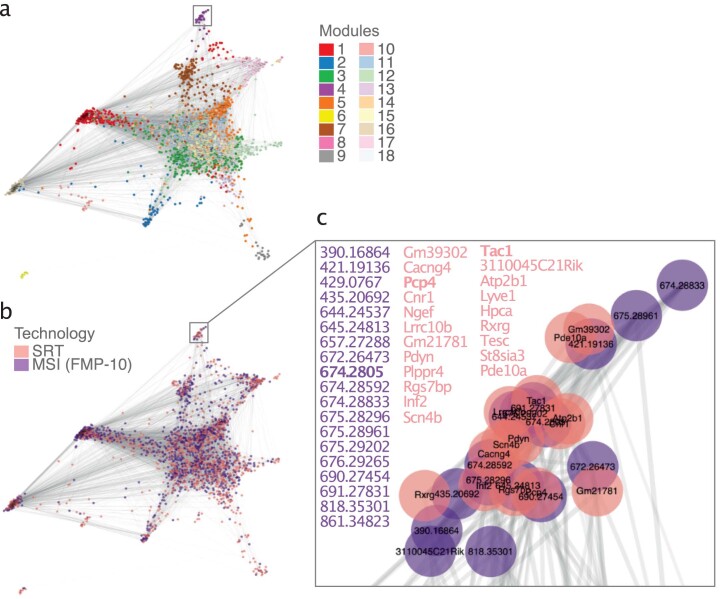
Joint embedding of gene and FMP-10 MSI peaks. Force directed layout of 10K-nearest neighbors was run on cosine distance graph (represented by edge thickness and shade). Nodes (genes and peaks) were split in 18 spatially co-detected modules using the Spinglass algorithm (**a**) and by technology of origin (**b**). Genes and peaks whose expression were associated with dopamine (m/z 674.2805) were enriched in module 4 and shown in (**c**).

To further demonstrate the applicability of SMA, we used it on a mouse model of Parkinson’s disease (PD). PD is the most common neurodegenerative disorder among the human population after Alzheimer’s disease^10,11^. It is characterized by the loss of dopaminergic neurons within the substantia nigra pars compacta (SNc), containing neurons that project to the dorsal putamen of the striatum^12^. In the present study, we aimed to capture both gene expression and corresponding neurotransmitters from two brain regions (SNc and striatum) of three unilateral 6-hydroxydopamine (6-OHDA)-lesioned mice^13^ (Fig. 2a,b (i and vi) and Extended Data Fig. 5). As expected, SMA predominantly detected dopamine in the intact striatum and SNc, but not in the lesioned contralateral hemisphere (Fig. 2b (ii and vii)). The multimodal data produced through SMA was also leveraged to identify the expression of genes associated with dopamine expression. Unsurprisingly, the key dopaminergic pathway genes *Th*, *Slc6a3*, *Slc18a2* and *Ddc* were found to be correlated with dopamine expression in the SNc^14^. Likewise, in the striatal sections, the dopamine levels were positively correlated with the expression of genes like *Pcp4* and *Tac1* and negatively correlated with genes like *Penk* and *Cartpt*, suggesting dysregulation of medium spiny neurons (MSNs; Fig. 2b (iv and ix) and 2d (i and ii) and Extended Data Figs. 6 and 7), while corroborating previous findings^15–17^. To substantiate our results, we sought to perform cell-type deconvolution^18^ using scRNA-seq data from a mouse brain atlas^19^. Strikingly, we found a lower proportion of midbrain dopaminergic neurons MBDOP2 in the lesioned SNc and ventral tegmental area (VTA). A similar phenotype was observed in the lesioned dorsal striatum for MSN1 neurons, a subtype of MSNs (Fig. 2b (v and x)). Furthermore, we were able to specify the localization of multiple neurotransmitters and metabolites, such as taurine, 3-methoxytyramine, 3,4-dihydroxy-phenylacetaldehyde (DOPAL), 3,4-dihydroxyphenylacetic acid, norepinephrine, serotonin, histidine, tocopherol and gamma-aminobutyric acid (Extended Data Fig. 5). The results demonstrated a similar spatial distribution of molecules as was previously reported in rat models using MALDI-MSI alone^8^.

**Extended Data Fig. 8:**
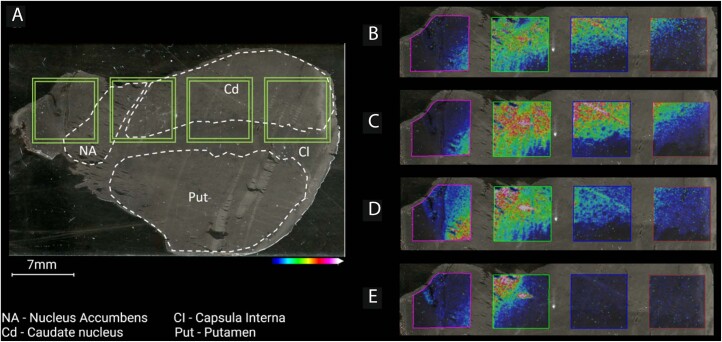
A scanned image of the human striatal tissue section on a Visium glass slide. (**a**) Whole tissue scan with annotated brain regions, where green squares indicate the areas coated with oligonucleotides. Ion images of **(b)** dopamine, **(c)** 3-MT, **(d)** serotonin, and **(e)** norepinephrine (double derivatized). All ion distributions are scaled to 50% of the maximum intensity and are all displayed as single derivatized species, unless otherwise stated.

**Extended Data Fig. 9:**
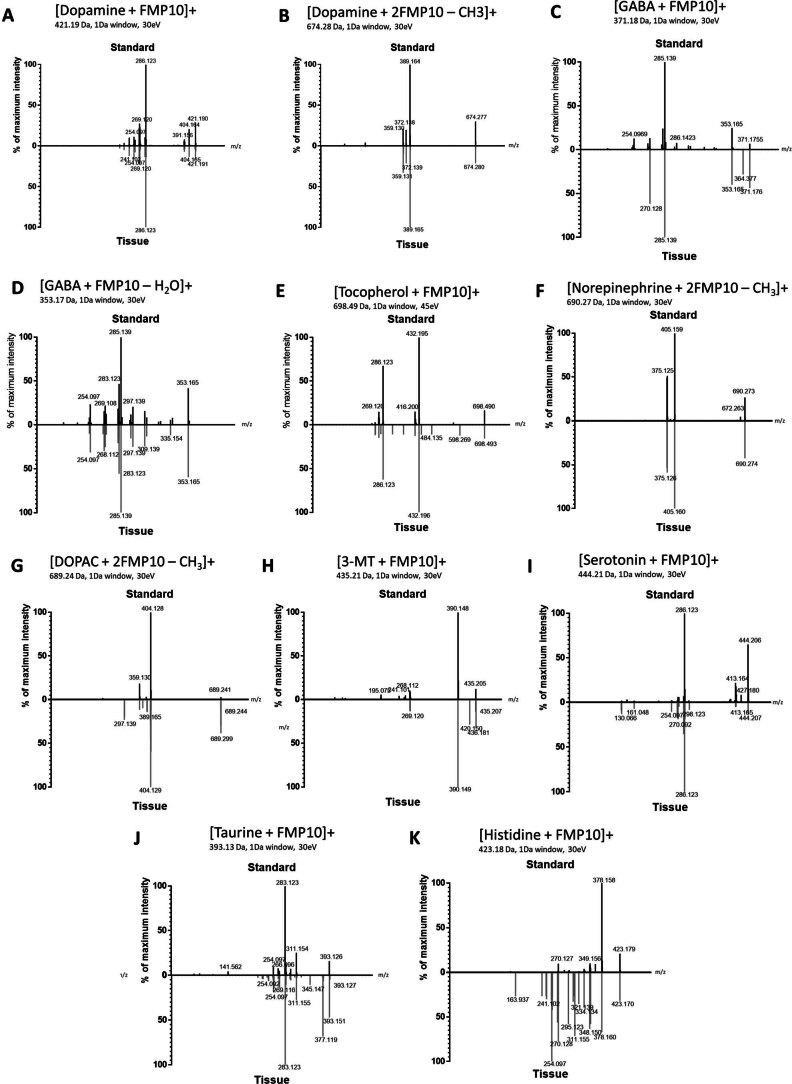
Mirror plots showing MALDI-MS/MS identification of FMP-10 derivatized metabolites. Mirrored spectra obtained from standards and mouse tissue sections derivatized with FMP-10, respectively. (**a**) Dopamine, m/z 421.19, (**b**) dopamine double derivatized, m/z 674.28, (**c**) GABA, m/z 371.18, (**d**) GABA-H20, m/z 353.17, (**e**) tocopherol, m/z 698.49, (**f**) norepinephrine double derivatized, m/z 690.27, (**g**) DOPAC double derivatized, m/z 698.24, (**h**) 3-MT, m/z 435.21, (**i**) serotonin, m/z 444.21, (**j**) taurine, m/z 393.13, and (**k**) histidine, m/z 423.18.

To demonstrate the relevance of the presented multimodal approach in human specimens, we applied it to a frozen human PD postmortem striatal brain sample and measured neurotransmitters and gene expression over a 2.4 × 0.5 cm tissue section (Fig. 2c (i)). To address and overcome RNA degradation in postmortem material, we applied a recent protocol specifically developed for gene expression measurements from fresh frozen tissue samples with low/moderate RNA quality^20^. The spatial MSI distribution of DA and 3-methoxytyramine (Fig. 2c (ii) and Extended Data Figs. 8 and 9) confirmed our previous findings^8^ in that these neurotransmitters were observed at higher levels in the medial division of the ventral caudate nucleus. Multimodal correlation analysis identified *SCG2* as the transcript that was most correlated with dopamine abundance (Fig. 2c,d (iv and iii, respectively)). We performed cell-type deconvolution analysis using publicly available snRNAseq data from the caudate nucleus of human postmortem samples^21^, which contains six clusters of MSN neurons (MSN.D1a-c and MSN.D2a-c). Interestingly, similar to our observation in the PD model, MSN.D1b neurons were enriched in the dopamine-expressing region, with a spatial pattern similar to *SCG2* (Fig. 2c (v)).

To summarize, we present a method that enables the simultaneous spatial profiling of small molecules and gene expression within a tissue section. Our approach also holds promise for fixed samples and additional modalities such as tagged antibodies^22,23^. The impact of this technology could be extended to other disciplines including oncology. SMA of tumor samples could provide new insights into the dynamic crosstalk that regulates the tumor microenvironment^24^ and drives the response to treatment^25^. Gene expression can also be leveraged to infer genomic integrity^26^, which is important to matching tumor clones with drug efficacy. The presented approach provides a further level of multimodality when studying small molecules in a tissue context.

## Methods

### Animal experiments

A total of four adult male C57Bl/6J mice, 8 weeks old (Charles River Laboratories), were housed under controlled temperature and humidity (20 °C, 53% humidity) with 12 h light/12 h dark cycles. The mice had access to standard food pellets and water ad libitum. All the animal work was performed in agreement with the European Council Directive (86/609/EE) and approved by the local Animal Ethics Committee (Stockholms Norra Djurförsöksetiska Nämnd, 3218-2022).

During the experiments, one mouse served as the control while three mice were anesthetized with isoflurane (Apoteket), pretreated with 25 mg kg^−1^ desipramine intraperitoneally (i.p.; Sigma-Aldrich) and 5 mg kg^−1^ pargyline i.p. (Sigma-Aldrich), placed in a stereotaxic frame and injected over 2 min, with 3 μg of 6-OHDA in 0.01% ascorbate (Sigma-Aldrich) into the median forebrain bundle (MFB) of the right hemisphere over two min. The coordinates for injection were anterior–posterior (−1.1 mm), medial–lateral (−1.1 mm) and dorsal–ventral (−4.8 mm) relative to bregma and the dural surface^27^. During the postoperative phase, the analgesic buprenorphine (Temgesic, 0.1 mg kg^−1^) was subcutaneously administered for 2 d following surgery. Two weeks after unilateral 6-OHDA administration, the lesion was validated by administering the mice with 1 mg kg^−1^ apomorphine i.p. (Sigma-Aldrich) and assessing rotational behavior in the mice. After the mice were sacrificed, the brains were removed, collected and stored at −80 °C for further use.

Efforts were taken to minimize the number of animals used and their suffering. Animals were killed by decapitation, and brains were rapidly removed, snap-frozen in dry-ice cooled isopentane for 3 s and stored at −80 °C to minimize postmortem degradation.

### Human postmortem sample

The human postmortem sample was from the caudate-putamen level of the brain (coronal sections) of a man who died at 94 years of age. The postmortem interval until the brain was frozen was 9.25 h. The neuropathological diagnosis was PD in Braak stage 3. The case was obtained from the Harvard Brain Tissue Resource Center at the McLean Hospital (Belmont, MA). Analyses were approved by the local ethical committee (Karolinska Institutet, Stockholm, Sweden, 2014/1366-31). All experiments were performed in compliance with all relevant ethical regulations.

### Tissue processing and sample preparation

Coronal mouse brain tissue sections, 12 μm thick, were cut at −20 °C using a CM1900 UV cryostat-microtome (Leica Microsystems) and subsequently thaw-mounted onto Visium glass slides (10X Genomics; for SMA and Visium analysis) or conductive indium tin oxide (ITO)-coated glass slides (Bruker Daltonics; for MSI analysis). For SMA experiments, Visium Gene Expression or Visium Tissue Optimization glass slides were used in their native form, without applying any modification. Sections were collected at the striatal level (distance from bregma, 0.49 mm^27^) and at the substantia nigra level (distance from bregma −3.39 mm). The human striatal PD sample was sectioned at 10 µm thickness, and the caudate region was placed over the four capture areas of the Visium slide (Extended Data Fig. 8). The prepared slides were stored at −80 °C. Sections were desiccated at room temperature for 15 min before scanning on a flatbed scanner (Epson Perfection V500), with the exception of tissues coated with FMP-10, which were scanned after matrix application. For neurotransmitter analysis, on-tissue chemical derivatization was performed with the FMP-10 reactive matrix according to a previously described protocol^8^. Briefly, a freshly prepared solution of FMP-10 (4.4 mM) in 70% acetonitrile was sprayed onto mouse brain tissue sections and the human tissue sample over 20 passes at 90 °C using a robotic sprayer (TM-Sprayer; HTX Technologies) with a flow rate of 80 μl min^−1^, spray head velocity of 1,100 mm min^−1^, 2.0 mm track spacing and 6 psi nitrogen pressure. Tissue sections from the control mouse and from lesioned mice were also coated with either 9-AA (5 mg ml^−1^ dissolved in 80% methanol; for analysis in negative ionization mode) or DHB (35 mg ml^−1^ dissolved in 50% acetonitrile and 0.2% trifluoroacetic acid; for analysis in positive ionization mode). 9-AA was applied using the TM-sprayer (75 °C, six passes, solvent flow rate of 70 μl min^−1^, spray head velocity of 1,100 mm min^−1^ and track spacing of 2.0 mm), while DHB was applied using the same settings, except for a nozzle temperature of 95 °C. Norharmane (7.5 mg ml^−1^ in 80% MeOH) was sprayed in 16 passes using the TM-sprayer with the following parameters: temperature 60 °C, flow rate of 70 μl min^−1^, spray head velocity of 1,200 mm min^−1^, 2.0 mm track spacing and 6 psi nitrogen pressure. Tissue sections mounted on the same glass slide but coated with different matrices were masked using a glass cover slip.

### Statistics and reproducibility

Eighteen sections originating from the same bregma level were used for the technical analysis (Fig. 1 and Supplementary Data) and processed according to the following four different protocols: MALDI-MSI, Visium, Visium internal control (V-iCTRL) and SMA. Nine of these sections were mounted on three ITO conductive slides (three sections per slide, one from each mouse) and were processed with MALDI-MSI protocol over three independent experiments. The remaining nine sections were mounted on three Visium Gene Expression Slides and were processed with Visium, V-iCTRL or SMA protocol over two independent experiments, one for the V-iCTRL and SMA sections and one for the VISIUM sections. We labeled the sections with a short identifier composed of the following: (1) the protocol (MSI for MALDI-MSI, VISIUM for Visium, V-iCTRL for Visium internal control, or SMA followed by 9-AA, FMP-10 or DHB for samples analyzed with SMA and sprayed with one of those matrices); (2) an acronym of the sample (m1, m3 or m4) and (3) a serial number specific for each tissue section. Sections labeled as V-iCTRL are sections where MALDI-MSI was not performed, but they are placed on an array where MALDI-MSI was performed on other sections of the same array. The V-iCTRL was performed with and without the application of a matrix—in the first case, the name of the matrix was added to the acronym of the sample (V-iCTRL.FMP10.m3.8), whereas in the second case, it was not (V-iCtrl.m3.3). Sections that were processed following the Visium protocol recommended by the manufacturer (10X Genomics) are labeled as VISIUM.

The results shown in Fig. 2b come from two representative sections from the striatum (panels ii–v) and substantia nigra (panels vii–x) of the mouse PD model and were analyzed over two independent experiments. In particular, for the striatum level, the sections SMA.FMP-10.mPD1.5, SMA.FMP-10.mPD3.6 and SMA.FMP-10.mPD4.7 already included in the technical analysis were analyzed. Three additional substantia nigra sections, one from each animal and placed on the same Visium array, were processed with SMA.FMP-10 protocol over one different independent experiment. Similar results were obtained from each of the three sections of the two groups.

Detailed information about the experimental design of our study, including independent experiments and replicates, can be found in Supplementary Data.

### MALDI-MSI

Tissue sections were imaged at 100 µm lateral resolution using a MALDI–Fourier-transform ion cyclotron resonance (7T solariX XR-2Ω, Bruker Daltonics) instrument equipped with a Smartbeam II 2 kHz Nd:YAG laser. Laser power was optimized at the start of each analysis. Spotted red phosphorus was used for external calibration of the methods. Spectra were collected by compiling the signals from 100 laser shots per pixel. Samples coated with FMP-10 and DHB were analyzed in positive ionization mode. The quadrupole isolation *m/z* ratio (Q1) was set at *m/z* 379 (FMP-10) or *m/z* 150 (DHB), and data were collected for samples coated with FMP-10 and DHB over the *m/z* 150–1,050 and *m/z* 129–1,000 ranges, respectively. For the FMP-10 analysis, *m/z* 555.2231 was used as the lock mass and the matrix peak at *m/z* 273.0394 was used as the lock mass for internal *m/z* calibration of the data acquired from the DHB-coated sample. Samples coated with 9-AA were analyzed in negative ionization mode over the *m/z* 107.5–1,000 range with a Q1 of *m/z* 120, and *m/z* 193.0771 was used as the lock mass. Lipid analysis with norharmane was performed in both positive and negative modes. Positive mode data were collected in the *m/z* range 150–1,200, Q1 *m/z* 250 and lock mass *m/z* 798.540963 ((PC(32:1))K^+^). Negative mode data were collected in the *m/z* range 150–2,000, Q1 *m/z* 220 and lock mass *m/z* 885.549853 ((PI(38:4))H^−^). Immediately after MSI analysis, the tissue-containing glass slides with tissue samples were washed twice for 30 s in prechilled methanol and then stored at −80 °C until Visium Gene Expression/Tissue Optimization processing.

### Molecule identification

All presented metabolites were identified by initial accurate mass matching with <1 ppm accuracy and subsequent MS/MS analysis. MALDI-tandem MS (MS/MS) was performed on tissue samples by acquiring spectra from brain regions in which the target ion was abundant. Endogenous metabolites that were derivatized with FMP-10 were identified by comparing their product ion spectra to those of corresponding derivatized standards (Extended Data Fig. 9). This comparison was performed in all cases except for DOPAL where no standard was available. DOPAL was found to colocalize to its precursor DA and other dopamine metabolites, and its localization was also confirmed using an additional derivatization strategy, that is, the 1-(4-(aminomethyl)phenyl)pyridin-1-ium chloride^28^, that covalently charge-tag molecules containing carboxylic acids and/or aldehydes. Validation data will be provided upon request. For nonderivatized metabolites, their MS/MS spectra were compared to reference spectra available online, such as those found at https://pubchem.ncbi.nlm.nih.gov or www.lipidmaps.org (Extended Data Fig. 9). The isolation window and collision energies used for individual metabolites are reported in Supplementary Data.

### MALDI-MSI and SMA metabolomics data analysis

The ftmsControl (Bruker Daltonics, v 2.3.0) and the flexImaging (Bruker Daltonics, v 5.0) software were employed for data acquisition. Subsequently, the SCiLS Lab Pro (Bruker Daltonics, v 2023b) and the SCiLS Lab API (Bruker Daltonics, v 6.0) software were used to generate ion images intended for downstream analyses. To ensure similar *m/z* lists among samples with different derivatization matrices, a reference peak list was used to calibrate all of the samples with the same derivatization matrix; thus, there was only one list of *m/z* values per matrix. To measure the MSI performance on Visium and ITO glass slides, Pearson’s correlations of mean spectra from consecutive sections analyzed on ITO or Visium were calculated using the SCiLS Lab API and the Python programming language (v 3.10).

### Visium Spatial Gene Expression and Tissue Optimization

FF samples were cryo-sectioned at 12 µm thickness, mounted onto Visium glass slides and stored at −80 °C before processing. Spatial gene expression libraries were generated following 10X Genomics Visium Gene Expression and Tissue Optimization protocols according to the manufacturer’s recommendations (Visium Spatial Gene Expression Reagent Kits—Tissue Optimization User Guide, document CG000238 Rev E, 10X Genomics, (February 2022); Visium Spatial Gene Expression Reagent Kits—User Guide, document CG000239 Rev F, 10X Genomics, (January 2022) and Methanol Fixation, H&E Staining and Imaging for Visium Spatial Protocols, document CG000160 Rev C, 10X Genomics). Libraries were sequenced using a NextSeq2000 sequencing system (Illumina). The length of read 1 was 28 bp, while the length of read 2 was 150 bp long.

### SMA

Sections of the Visium Gene Expression or Tissue Optimization glass slides (10X Genomics) were desiccated at room temperature for 15 min before the reactive matrices were applied.

Matrix application and MSI were performed as already described in the section MALDI-MSI. After MSI, the slides were briefly immersed in prechilled methanol (2 × 30 s), followed by storage at −80 °C until Visium Gene Expression/Tissue Optimization was performed. Visium Spatial Gene Expression and Tissue Optimization slides, with the exception of the human postmortem sample, were processed according to the corresponding latest versions of the 10X Genomics protocols (Visium Spatial Gene Expression Reagent Kits—Tissue Optimization User Guide, document CG000238 Rev E, 10X Genomics, (February 2022); Visium Spatial Gene Expression Reagent Kits—User Guide, document CG000239 Rev F, 10X Genomics, (January 2022) and Methanol Fixation, H&E Staining and Imaging for Visium Spatial Protocols, document CG000160 Rev C, 10X Genomics), without any modification. Libraries were sequenced using the Nextseq2000 sequencing system (Illumina). The length of read 1 was 28 bp, while the length of read 2 was 150 bp long. The human postmortem sample was processed according to the RRST protocol^18^.

### Visium data processing

Sequenced libraries were processed using Space Ranger software (v 1.2.1 for standard Visium data and v 1.3.1 for RRST data; 10X Genomics). Reads were aligned to the prebuilt human or mouse reference genome, including a GTF file, a fasta file and a STAR index, provided by 10X Genomics (GRCh38 for human data or mm10 for mouse data, v 32, Ensembl 98). Gene body coverage analysis was performed using the possorted_genome_bam.bam files included in the Space Ranger output as input to RSeQC (v 5.0.1)^29^. Analyses of sequencing saturation and median genes per spot as functions of mean reads per spot were performed using the values provided in the 'web_summary.html' files from the Space Ranger output.

### Visium, RRST and SMA transcriptomics data analysis

Minimum, maximum, mean and s.d. of the fluorescence intensities from the Visium Tissue Optimization experiment were extracted using the software ImageJ (v 1.53)^30^ and plotted using R (v4.1.3) and the R package ggplot2. The spatial transcriptomics data obtained with either standard Visium, RRST or SMA were processed and analyzed using R (v 4.1.3), the single-cell genomics toolkit Seurat and the spatial transcriptomics toolkit STUtility. The H&E images were manually annotated based on tissue morphology and dopamine expression using the interactive application Loupe Browser (10X Genomics, v 6.3.0). Mouse striatum and substantia nigra hemispheres were categorized into two groups, that is, ‘intact’ for the left hemisphere and ‘lesioned’ for the right. The filtered count matrices obtained from spaceranger were used in subsequent analysis upon application of additional filters. In particular, spots below sectioning or mounting artifacts were annotated using Loupe Browser (v 6.3.0) and removed using the ‘SubsetSTData’ function in STUtility; spots that included more than 38% mitochondrial genes or less than 50 unique genes were removed using the same STUtility function; hemoglobin-coding, riboprotein-coding and Malat1 genes were removed from the dataset as well. Pearson correlation coefficients and *P* values were calculated using the corrplot function of the corrplot R package. After filtering out spots and genes as previously described, the data were normalized and subjected to a basic analytical workflow using functions from the Seurat R package. The SCTransform function was used for normalization and variance stabilization and was followed by dimensionality reduction via PCA (RunPCA). Data were integrated with the RunHarmony function from the harmony R package using group.by.vars = ‘Sample.ID’ (which indicates the sample of origin), assay.use = ‘SCT’ and reduction = ‘pca’ as parameters. A shared nearest neighbor graph was constructed based on the first 30 principal components (FindNeighbors). Finally, a uniform manifold approximation and projection (UMAP) embedding was computed based on the first 30 principal components (RunUMAP); this was followed by graph-based clustering (FindClusters). Marker genes for each identified cluster were calculated using the function FindAllMarkers with default parameters, while a nonparametric Wilcoxon rank-sum test and the Bonferroni correction were used for *P* value adjustment. Only genes with log_2_ fold change higher than 0.25 and adjusted *P* value lower than 0.01 were considered differentially expressed. MSI and SRT were manually aligned using the interactive Shiny application (available in STUtility) through the function ManualAlignImages. The alignment procedure requires images of the tissue sections to be aligned, that is, one image for the RNA data and one image for the MSI, as input. However, because we only had access to H&E images for the RNA data, we modified the alignment procedure to align the MSI data points directly to the corresponding H&E image. Before alignment, the MSI data first underwent PCA to identify and remove data points that were located outside of the tissue sections. Once the RNA data had been aligned to the MSI data, we identified pairs of nearest neighbors across the two datasets using the k-nearest neighbors function from the dbscan R package, with *k* set to 5. To remove parts of the tissue sections that did not overlap in the two datasets, we filtered out pairs with a distance higher than 35 pixels, a threshold that was empirically determined from the histogram of neighbor distances. Next, only the closest neighbor in the RNA data was kept for each MSI data point, thus generating a list of MSI-RNA data point pairs. As MSI yielded a lower density of data points than Visium, we decided to select neighbors for MSI data points rather than the other way around. In cases where the multiple MSI data points shared the same nearest neighbor, this neighbor was reused to ensure a one-to-one mapping. Once the MSI-RNA data point pairs had been identified, we used these pairs to subset the raw data and produce new Seurat objects with the two aligned data modalities stored in separate assays. Identification of the genes that were most correlated with dopamine levels was performed by calculating pairwise Pearson correlation coefficients between dopamine and all of the genes retained in the dataset. *P* values were adjusted using Benjamini and Hochberg correction method, and only genes with a false discovery rate (FDR) lower than 0.01 were retained. Cell-type proportions were inferred using stereoscope, a probabilistic method designed to deconvolve spatial data using single-cell data. These analyses were run in accordance with the developer’s recommendations (https://github.com/almaan/stereoscope), using the 5,000 most highly variable genes, based on the FindVariableFeatures function from the Seurat R package. The spatial transcriptomics data of the mouse model was deconvolved using scRNA-seq data from the mouse brain atlas (http://mousebrain.org/adolescent/). Cells occurring more than once in the single-cell count matrix were removed from the dataset. The 39 annotated taxa were used as a basis for deconvolution, along with the four annotated clusters of dopaminergic neurons and the six annotated clusters of MSNs present in the dataset. In total, 50,000 epochs were used for both single-cell and spatial transcriptomics data, and the batch sizes were set to 2,048. Single-cell data were subsetted to a maximum of 1,000 cells per cell type, using the --sc_upper_bound option. The spatial transcriptomics data of the human sample were deconvolved using snRNAseq data from GSE178265 repository. This dataset was filtered to retain only caudate nucleus samples, high-quality nuclei (number of genes between 1,000 and 10,000, number of unique molecular identifiers lower than 50,000, mitochondrial genes content lower than 7%) and a subsample of 4,000 cells per donor. The data were then normalized and subjected to a basic single-cell analysis workflow. The functions SCTransform and RunPCA from the Seurat R package were used for normalization and variance stabilization and dimensionality reduction, respectively. Data were integrated with the RunHarmony function from the harmony R package using group.by.vars = ‘orig.ident’ (which indicates the sample of origin), assay.use = ‘SCT’ and reduction = ‘pca’ as parameters. The functions FindNeighbors, RunUMAP and FindClusters from the Seurat R package were used to construct a shared nearest neighbor graph, compute a UMAP embedding and perform graph-based clustering. Using a resolution of 0.6, a total number of 23 clusters were detected and used for cell-type deconvolution. In total, 75,000 epochs were used for both single-cell and spatial transcriptomics data, and the batch sizes were set to 100. Single-cell data were subsetted to a maximum of 250 and a minimum of 25 cells per cell type, using the -sub and -slb options, respectively. After deconvolution, the cell-type proportion values were overlaid on the tissue section images by using the FeatureOverlay function in the STUtility package.

### Peak-associated genes ranking

Identification of genes whose expression is associated with metabolite peaks was done using k-nearest neighbors graphs on the top 2,000 high-variable genes. Briefly, sections processed with SMA-FMP-10 were grouped based on their bregma level (striatum or substantia nigra) to (1) perform joint dimensionality reduction of the two modalities using PCA, (2) then built a binary search tree using the PCA co-embedding of the SRT data using cosine similarity (3) and finally search the ten nearest neighbors for each peak. The resulting graph containing indexes and distances was then annotated to contain gene and peak names, available in Supplementary Data. Integrated graphs were used for the identification of MSI peak and gene spatially codetected modules using the Spinglass community detection algorithm, which is suitable for nonsymmetric graphs. Visualization of the gene-peak neighborhood was done using a weighted Distributed Recursive Layout.

### Targeted in situ sequencing sample pretreatment

Post-MALDI imaging, the mouse coronal section was fixed with 4% formaldehyde for 5 min, followed by permeabilization with 0.1 M HCl for 5 min. The section is then dehydrated in an ethanol series of 70% and 100% for 2 min each.

### Targeted in situ sequencing protocol

PLP hybridization was performed overnight at 37 °C with 10 nM final concentration of phosphorylated padlock probes in PLP hybridization buffer (2× SSC, 10% formamide). Sections were then washed 2× with PLP hybridization buffer and 2× with PBS. Ligation was performed for 2 h at 37 °C with 1× T4 Rnl2 reaction buffer (NEB B0239SVIAL), 1 U µl^−1^ T4 Rnl2 (NEB, M0239), 1 U μl^−1^ RNase inhibitor (BLIRT, RT35) and rolling circle amplification (RCA) primer at a final concentration of 50 nM. The sections were washed twice with PBS before proceeding with rolling circle amplification at 30 °C, overnight with 0.5 U μl^−1^ Φ29 polymerase (Monserate Biotech, 4002) in reaction mixture of 1× Φ29 buffer (50 mM Tris–HCl, 10 mM MgCl_2_ and 10 mM (NH_4_)_2_SO_4_), 5% glycerol, 0.25 mM dNTPs (BLIRT, RP65) and 0.2 μg μl^−1^ BSA.

Fluorescent probe detection was performed by hybridization of readout detection probes (100 nM) and DAPI (Biotium, S36936) in a hybridization buffer (2× SSC, 20% formamide) for 45 min at room temperature. Sections were washed with PBS and mounted with SlowFade Gold Antifade Mountant (Thermo Fisher Scientific, S36936).

### Imaging of targeted in situ sequencing

All images were obtained with a Leica DMi8 epifluorescence microscope equipped with an external LED light source (Lumencor SPECTRA X light engine), automatic multislide stage (LMT200-HS), sCMOS camera (Leica DFC9000 GTC), and objectives (HC PL APO ×10/0.45; HC PL APO ×20/0.80; HCX PL APO ×40/1.10 W CORR). Multispectral images were captured with a microscope equipped with filter cubes for six dye separation and an external filter wheel (DFT51011). Image scanning was performed with 10% tiled image overlap. Z-stack imaging of 10 µm at 1.0 µm steps to cover the depth of the tissue. The images for analysis were obtained from Leica DMi8 microscope with LASX software (v 3.7.5.24914) and raw image files were exported for analysis.

### Padlock probe sequences

The padlock probe sequences file is made available for the reader in the subfolder in situ sequencing of the data repository.

### In situ sequencing data analysis

Targeted in situ sequencing—images from targeted in situ sequencing experiment were analyzed on a custom MATLAB script on MATLAB (R2019b.) Intensity measurements over RCPs—for each channel image, the RCPs intensity was measured over the RCP, ten pixels in each direction from the middle, which generated a total of 21 intensity measurements. The mean maximum intensity of RCPs was then calculated across the control and MALDI-MSI imaged area.

### Reporting summary

Further information on research design is available in the Nature Portfolio Reporting Summary linked to this article.

## Online content

Any methods, additional references, Nature Portfolio reporting summaries, source data, extended data, supplementary information, acknowledgements, peer review information; details of author contributions and competing interests; and statements of data and code availability are available at 10.1038/s41587-023-01937-y.

### Supplementary information

Reporting Summary

Supplementary Data Supplementary Table 1: Table illustrating the type of glass and matrices used for the samples included in Fig. 1b. Supplementary Table 2: Metadata of all the samples included in the article. Supplementary Tables 3–19: MALDI-MS/MS identification of metabolites mentioned in the article. Supplementary Tables 20–23: Results of gene and peaks joint embedding analysis (Extended Data Fig. 7). Supplementary Tables 20 and 21: Top ten genes related to each FMP-10 peak in the mouse striatum and substantia nigra. Supplementary Tables 22 and 23: The results of spinglass clustering.

## Data Availability

The databases GRCh38 for human data and mm10 for mouse data, v 32, Ensembl 98 were used for the alignment of the sequencing data. All data required to replicate the analyses, including spaceranger output files, H&E images and additional files are available at Mendeley Data (10.17632/w7nw4km7xd.1). The MALDI-MSI raw data in imzML format and MS/MS spectra collected from tissue in mzML and vendor-specific baf format are available at Figshare (10.17044/scilifelab.22770161). In situ sequencing data are available at Zenodo (10.5281/zenodo.7861508). Spatial transcriptomics raw sequencing data, microscope images and spaceranger output files of the mouse and human samples are available at Figshare (10.17044/scilifelab.22778920). The same data, with the exception of the human sequencing data, is available at Gene Expression Omnibus (GEO) with the accession number GSE232910. The sequencing data in FASTQ format of the human sample is provided upon request by Per Svenningsson (per.svenningsson@ki.se). The mouse brain atlas (http://mousebrain.org/) dataset and the GEO repository with accession number GSE178265 were used for the ST data deconvolution.

## Code availability

The code used to generate the figures is available at https://github.com/marcovito/sma.
