# Supplementary material for: Spatial multimodal analysis of transcriptomes and metabolomes in tissues
Source: Nat Biotechnol. 2023 Sep 4;42(7):1046–50. doi: 10.1038/s41587-023-01937-y (PMC11251988; doi:10.1038/s41587-023-01937-y)
Supplement: Supplementary file 1 — Reporting Summary [file 41587_2023_1937_MOESM1_ESM.pdf]

nature portfolio

Corresponding author(s): Joakim Lundeberg

Last updated by author(s): May 17, 2023

Reporting Summary

Nature Portfolio wishes to improve the reproducibility of the work that we publish. This form provides structure for consistency and transparency in reporting. For further information on Nature Portfolio policies, see our Editorial Policies and the Editorial Policy Checklist.

Statistics

For all statistical analyses, confirm that the following items are present in the figure legend, table legend, main text, or Methods section.

n/a	Confirmed	
☐	☒	The exact sample size (*n*) for each experimental group/condition, given as a discrete number and unit of measurement
☐	☒	A statement on whether measurements were taken from distinct samples or whether the same sample was measured repeatedly
☐	☒	The statistical test(s) used AND whether they are one- or two-sided<br>*Only common tests should be described solely by name; describe more complex techniques in the Methods section.*
☐	☒	A description of all covariates tested
☐	☒	A description of any assumptions or corrections, such as tests of normality and adjustment for multiple comparisons
☐	☒	A full description of the statistical parameters including central tendency (e.g. means) or other basic estimates (e.g. regression coefficient) AND variation (e.g. standard deviation) or associated estimates of uncertainty (e.g. confidence intervals)
☐	☒	For null hypothesis testing, the test statistic (e.g. *F*, *t*, *r*) with confidence intervals, effect sizes, degrees of freedom and *P* value noted<br>*Give P values as exact values whenever suitable.*
☒	☐	For Bayesian analysis, information on the choice of priors and Markov chain Monte Carlo settings
☐	☒	For hierarchical and complex designs, identification of the appropriate level for tests and full reporting of outcomes
☐	☒	Estimates of effect sizes (e.g. Cohen's *d*, Pearson's *r*), indicating how they were calculated

Our web collection on statistics for biologists contains articles on many of the points above.

Software and code

Policy information about availability of computer code

Data collection	MALDI-MSI data: FTMS control (Bruker Daltonics, version 2.3.0).<br><br>All spatially resolved transcriptomics data generated in this study was processed from raw sequencing files in FASTQ format using the 10x Genomics Space Ranger software (v 1.2.1 for standard Visium data and v 1.3.1 for RRST data, 10x Genomics).<br><br>Targeted in situ sequencing data: images for analysis were obtained from Leica DMi8 microscope with LASX software (version 3.7.5.24914) and raw image files were exported for analysis.
Data analysis	MALDI-MSI data: Ion images were created and analysed using the SCiLS Lab API (v 6.0 , Bruker Daltonics), the SCiIS Lab Pro software (v 2023b, Bruker Daltonics), flexImaging (v 5.0, Bruker Daltonics) and the Python programming language (v 3.10).<br><br>Visium Tissue Optimization fluorescence images were processed using ImageJ (v1.53). Sequenced libraries were processed using Space Ranger software (version 1.2.1 for standard Visium data and version 1.3.1 for RRST data, 10x Genomics). Visium, RRST or SMA data were processed and analyzed using R (v4.1.3). Gene body coverage analysis was performed using RSeQC (v5.0.1). Annotation of Visium spots and manual tissue detection was performed using Loupe Browser (v 6.3.0). The processed data were analyzed using R (v4.1.3). The code used to generate the figures will be made available upon publication at the website https://github.com/marcovito/sma<br><br>Targeted in situ sequencing: images from targeted in situ sequencing experiment were analysed on a custom Matlab script on Matlab (R2019b.) All code and processed images are available at (https://zenodo.org/record/7861508#.ZEexZZFBwck).

For manuscripts utilizing custom algorithms or software that are central to the research but not yet described in published literature, software must be made available to editors and reviewers. We strongly encourage code deposition in a community repository (e.g. GitHub). See the Nature Portfolio guidelines for submitting code & software for further information.

Data

Policy information about availability of data

All manuscripts must include a data availability statement. This statement should provide the following information, where applicable:

- Accession codes, unique identifiers, or web links for publicly available datasets
- A description of any restrictions on data availability
- For clinical datasets or third party data, please ensure that the statement adheres to our policy

All the MALDI-MSI raw data (imzML files) are published at Figshare with DOIs 10.17044/scilifelab.22770161.

All the Visium raw data (FASTQ files, microscopy images, space ranger outputs) except the human sample FASTQ files are publilshed at Figshare with DOI 10.17044/scilifelab.22778920. The same data is published on GEO with the following accession number: GSE232910. Access to raw human sample (sequence data) will be available through controlled access principles following GDPR legislation through MTA with Karolinska Institute, Per Svenningsson (per.svenningsson@ki.se).

The ISS raw data are published at Zenodo with DOI 10.5281/zenodo.7861508.

All processed data required to replicate the analyses, including spaceranger output files, H&E images and additional files are available at Mendeley Data will be made available upon publication at the following URL: https://data.mendeley.com/datasets/w7nw4km7xd/draft?a=4a71c2b4-81a8-4c4e-8cb4-b099128f25c1.

Human research participants

Policy information about studies involving human research participants and Sex and Gender in Research.

Reporting on sex and gender	Sex and gender are not considered important for this methodological study.
Population characteristics	Population characteristics are not considered important for this study. The human post-mortem sample was obtained from the Harvard Brain Tissue Resource Center at the McLean Hospital, Belmont, MA, USA. The human sample was from the caudate-putamen of a man who died at 94 years of age. The postmortem interval until the brain was frozen was 9.25 hours. The neuropathological diagnosis was Parkinson´s disease in Braak stage 3.
Recruitment	No recruitment process was done in order to perform this study. In this methodological study, we aimed to demonstrate and benchmark our protocol on different tissue types. The human post-mortem sample was obtained from the Harvard Brain Tissue Resource Center at the McLean Hospital (Belmont, MA, USA).
Ethics oversight	Analyses on the human post-mortem sample were approved by the local ethical committee (Karolinska Institutet, Stockholm, Sweden, no. 2014/1366-31). All experiments were performed in compliance with all relevant ethical regulations.

Note that full information on the approval of the study protocol must also be provided in the manuscript.

Field-specific reporting

Please select the one below that is the best fit for your research. If you are not sure, read the appropriate sections before making your selection.

☒ Life sciences ☐ Behavioural & social sciences ☐ Ecological, evolutionary & environmental sciences

For a reference copy of the document with all sections, see nature.com/documents/nr-reporting-summary-flat.pdf

Life sciences study design

All studies must disclose on these points even when the disclosure is negative.

Sample size	No sample size calculation was performed, as this work focused on developing a new analytical approach. Animal sample size was selected to the point that minimizes the number of sacrificed animals but preserves the statistical power of quantitative work. Ethical concerns, sample availability and acquisition time were limiting factors for the number of samples that were chosen.
Data exclusions	Only the samples needed to produce the figures were used in the data analysis. The Visium slides TO1, V11L12-067 and V11T17-105 were used for initial pilot experiments and were not included in the published dataset. The Visium capture areas V11T17-101_A1, C1 and D1 were included in the published dataset but they were not needed for the data analysis.
Replication	MALDI-MSI, Visium and ISS are destructive techniques, meaning that replicate experiment can not be done on the same tissue sample. For this analysis we considered technical replicates consecutive sections of the same mouse brain analyzed with the same protocol, and biological replicates sections of the same bregma level from different mouse brains analyzed with the same protocol. We measured the reproducibility of our method calculating Pearson's correlations across a representative subset of our dataset and the gold standard technologies (MALDI-MSI performed on ITO conductive glass and Visium). All the details about replication are described in the methods and Supplementary tables. All replication were successful.

Randomization	No randomization was performed, as randomization of samples is not relevant for this study which focused on developing a new analytical approach. Samples were chosen based on the expectation that they could show alterations of the dopaminergic pathway (both in the mouse 6-OHDA model and the human post-mortem sample with Parkinson's Disease). Data analysis of all samples in this study was performed in groups according to the species of origin (human or murine) and the brain area of the tissue section (striatum or substantia nigra).
Blinding	Blinding is not relevant for this study, as it focused on developing a new analytical approach. Samples from the same tissue specimens were analyzed together in order to make fair comparisons and extract meaningful biological observations, as each tissue is composed of different cell types.

Reporting for specific materials, systems and methods

We require information from authors about some types of materials, experimental systems and methods used in many studies. Here, indicate whether each material, system or method listed is relevant to your study. If you are not sure if a list item applies to your research, read the appropriate section before selecting a response.

Materials & experimental systems

n/a	Involved in the study
☒	☐ Antibodies
☒	☐ Eukaryotic cell lines
☒	☐ Palaeontology and archaeology
☐	☒ Animals and other organisms
☒	☐ Clinical data
☒	☐ Dual use research of concern

Methods

n/a	Involved in the study
☒	☐ ChIP-seq
☒	☐ Flow cytometry
☒	☐ MRI-based neuroimaging

Animals and other research organisms

Policy information about studies involving animals; ARRIVE guidelines recommended for reporting animal research, and Sex and Gender in Research

Laboratory animals	A total of four adult male C57Bl/6J mice (species: mus musculus), 8 weeks old and were purchased from Charles River (Sulzfeld, Germany)
Wild animals	This study did not involve wild animals
Reporting on sex	In this study only male mice were used. Sex was not considered in this study. This work presents a method for obtaining MALDI-MSI and spatial transcriptomics data from the same tissue section. We showed its potential of detecting altered neurotransmitters and transcripts in 6-OHDA mice and a Parkinson's Disease post-mortem sample. For the validity of these observations the sex of the subjects is not relevant.
Field-collected samples	This study did not involve field-collected samples
Ethics oversight	All of the animal work was performed in agreement with the European Council Directive (86/609/EE) and approved by the local Animal Ethics Committee (Stockholms Norra Djurförsöksetiska Nämnd, no. 3218-2022).

Note that full information on the approval of the study protocol must also be provided in the manuscript.
